# General Medicine and Therapeutics

**Published:** 1894-01

**Authors:** 


### GENERAL MEDICINE AND THERAPEUTICS.

EDITED BY DR. LUTHER B. GRANDY.

### ERGOTINE AND GALLIC ACID IN HEMOPTYSIS.

Dr. Blaschko, in cases of hemoptysis where injections of ergotine were of no service, has often succeeded with the internal administration of gallic acid, in stopping the hemorrhage. Therefore, in recent years he has combined it with ergotine in copious pulmonary hemorrhages and with the best of results. He employed the following formula:

℞. Gallic acid, 1 gm. (grs. xv.)
Ergotine, 1 gm. (grs. xv.)
Distilled water,
Syrup of orange peel, aa. 25 gms. (ʒ vj.)
A teaspoonful every two hours.

Where there is a great deal of irritation and inclination to cough he employed the syrup of opium instead of the orange peel syrup, and in threatening cases he prescribed a teaspoonful every hour. As this remedy is entirely reliable he was able to dispense with the use of the ice-bag to the chest and injections of ergotine subcutaneously, which latter often lead to suppuration. Small pieces of ice were swallowed and cold milk ordered to be drunk with the greatest possible rest of body and especially of the organs of speech. *Philadelphia Medical and Surgical Journal.*

### SALICYLIC ACID LOCALLY IN ACUTE RHEUMATISM.

Dr. Bourget, of Lausanne (*Medical Neuigkeiten,* No. 32, 1893), has found from numerous experiments that a salve of salicylic acid is rapidly absorbed in articular rheumatism. The large joints are simply annointed with the salve and then covered with flannel. The following gave the best results as to the quantity absorbed:

Salicylic acid, }
Lanoline, } aa. gms. 10.
Ess. of turpentine, }
(℥ ijss.)
Benzoated lard, gms. 80.
(℥ ijss.)

A half hour after inunction a strong reaction of salicylic acid is to be obtained on examining the urine. For ten years he has treated all his cases of acute articular rheumatism with this salve, without the internal administration of the acid. His results were very good. The pain disappears a few hours after application; the swelling diminishes, as a rule, from the second day; the fever disappears completely between the third and the fifth day, and, finally, with this method one never observes the disagreeable side or after effects which are so frequent with the internal administration of the acid.—*Lancet Clinic.*

---

### CHEMICAL COMPOUNDS.

A large proportion of the recently introduced antipyretics and antiseptics, though almost in every instance advertised to be a "chemical compound," have been found to be simple *mixtures* of several medicinal chemicals, combined in varying proportions. Thus "*Anticol*" is a proprietary article introduced to the world as the "sovereignest thing" for febrile affections. It has been unmasked, and is found to consist of 75 per cent. of antifebrin, 17.5 per cent. of sodium bicarbonate, and 7.5 per cent. of tartaric acid. "*Anticylic acid,*" introduced as an antipyretic and anodyne, is reported to be nearly a mixture of *anti*pyrine and sali*cylic* acid. "*Antidiphtherin*" is a name given to a new preparation claimed to be a remedy for diphtheria, and which, upon examination, is found to contain potassium chlorate and a trace of ferric chloride. "*Antinervin*" (so called "salicyl-bromanilide"), lauded as an anodyne and nervine, consists, according to E. Ritsert, of a mixture of one part of ammonium bromide, one of salicyclic acid and two of antifebrin.

"*Antiseptin*" (so-called "zinc borothymol-iodide"), extolled as an antiseptic, is, according to Goldmann, a mixture of 85 parts of zinc sulphate, 2½ of zinc iodide, 2½ of thymol, and 10 of boric acid; it must not be confounded with "antisepsin," which is chemically paramono-bromphenyl-acet-amide, nor with "antisep*tol*," which is cinchonine iodo-sulphate. "*Aseptin*," an antiseptic introduced some years ago by Gahn, is said to consist of a mixture of boric acid and alum. "*Camphol*" is an antiseptic and anodyne, consisting of a mixture of camphor and salol in varying proportions. "*Exodyne*" is another antipyretic and anodyne, which, according to F. Goldmann, is nothing but a mixture of 90 parts of antifebrin, 5 of sodium salicylate and 5 of sodium bicarbonate. "*Lysol*" is a disinfectant of very complex and indefinite chemical composition. "*Phenolid*" is an antipyretic and anodyne found by analysis to be a mixture of 58 parts of antifebrin and 42 of sodium salicylate. "*Quickine*" is an antiseptic composed, according to the *Pharmaceutische Zeitung*, of one part of corrosive sublimate, 50 of carbolic acid and 50,000 of dilute alcohol. "*Somnal*," introduced as a hypnotic, is reported to be merely a solution of chloral and urethane in alcohol.—*Merck's Report.*

---

### DIAGNOSIS OF TYPHOID FEVER.

Dr. V. Filipovich, of Odessa, remarks that in all cases of typhoid fever under his notice during the last two extensive epidemics in Southern Russia, there has been a peculiar induration with yellowish orange tint in the prominent portion of the plantar and palmar surfaces, instead of the reddish color observed in healthy subjects, or the bluish tinge in cyanotics. This yellowish appearance is to be accounted for by the enfeebled action of the heart, the incomplete filling of the capillaries, and the dryness of the skin.

The sign is so constant and so well marked that Dr. Filipovich considers it as pathognomonic even in doubtful cases. That it is in no sense peculiar to the epidemics in Southern Russia is vouched for by Dr. Skibnevski, who observed the same in patients suffering from this disease in the vicinity of Moscow.—*Medical Age.*

### LET US HAVE A CABINET PLACE IN THE NATIONAL GOVERNMENT.

Dr. C. H. Hughes, of St. Louis, President of the American Medical Editors' Association, in his address at the Editors' Banquet in honor of the Pan-American Medical Congress, and published in the *Alienist and Neurologist*, said that "as a physician, a practioner of the healing art, a teacher of medicine in school and with journal, I dare to proclaim that the wisest and best thing this government can do, both for its present and future welfare, for its perpetuity and growth among the nations, the most powerful, most beneficent and grandest of governments, would be to create a Bureau of Sanitation not merely to keep out foreign epidemics of contagious diseases, but a psychical and physical sanitation of the many forms of disease of body and mind known to science and modern medical progress, and recognize the profession of medicine as it does that of law, of agriculture and arms, by giving the most distinguished and capable of its votaries a proper and deserving place in the cabinet of the nation."—*Times and Register*.

---

### THE KEELY CURE.

Two years ago the daily press engaged largely in spreading the supposed virtues of this business, and in their philanthropic efforts succeeded in making the chief beneficiary a very rich man.

This newspaper-made man felt that the Western Continent presented an altogether too limited field for the great benefit (?) he was called upon to perform for a class of defective weaklings, who are, unfortunately, too numerous in most communities. To spread himself still further over the face of the earth he crossed to the British Isles, where his newspaper reputation had already made the name of Keely and his cure a familiar one.

The editors of the London *Lancet* and the *Medical Press and Circular*, leading weekly medical journals, proceeded to investigate the cure business, and soon satisfied themselves of its fraudulent character, and so proclaimed it to their readers. Their action afforded the doughty Keely an opportunity to spread his fame by suing the proprietors for large sums as damages. Keely wauled his persecution and martyrdom in the prosecution of his philanthropic work, erstwhile continuing to sell his supposed cure rights to gullible dupes wherever they could be found.

The British Isles having been harrowed and raked for the usufruct, the poor man returned to his native land, leaving his court suits behind him, which, when called, his attorneys promptly asked for a dismissal at the plaintiff's costs, as it was not convenient for him to be present to press the suits, that were to be a balm and solace to his wounded feelings.

The Keely fad has had its day, has deluded its scores and hundreds of victims. It is now of the past, and will, like Perkins' tractors, furnish food for laughter for those who care to read up such literature, or give it a place in commencement and introductory lectures in medical colleges.

The fatherly hand of the Continental governments affords ample protection to the people from such newspaper vaunted businesses as that pursued by Keely and his kind.

Slowly and by degrees the people of the American States are being awakened to the necessity of a police protection from human vultures, who for sordid gain prey upon the ignorance and credulity of the people. Medical practice acts, State boards of health and State boards of medical examiners with police powers and functions will in due course of time prove themselves to be of invaluable service as educators as well as advisers and protectors of the people in regard to all matters pertaining to the healthful care of all the people.—*Cincinnati Lancet-Clinic.*

---

### COMPARATIVE INFLUENCE OF LARGE AND SMALL DOSES OF IRON.

The comparative effects of large and small doses of iron and the indications for the one or the other are discussed in the *Therapeutic Gazette.* The experience of the writer in treating twelve cases of anemia, which were as far as possible alike, confirmed his belief in the small doses. Six of these people received two or three grains of reduced iron three times a day, and the remaining six received one third of a grain three times daily. The six that received the small doses had far less disorder of digestion than those who received the large doses. They recovered as promptly as those receiving the large doses, if not more so. It is true that in some conditions in which there is gastro-intestinal disorder associated with the formation of gas arising from fermentation or decomposition, and in which the anemia is largely due to the destruction of the constituents of the blood by the absorption of poisonous materials from the intestine, large doses of iron are absolutely necessary, because in these instances only a small quantity of iron is absorbed, and the greater amount of it forms a sulphide of iron or other compound, with the contents of the intestine. Where we have, therefore, a destruction of the iron in large amount, it may be necessary to give it in full dose; but unless this be the case, one-eighth of a grain of reduced iron will in most cases give better results than three grains. Under these circumstances constipation more rarely occurs, and we also avoid in this way the so-called iron headache.—*Boston Medical and Surgical Journal.*

